# Increased prevalence of malignancy in adult mitochondrial disorders

**Published:** 2013-12-25

**Authors:** J Finsterer, E Krexner

**Affiliations:** * Krankenanstalt Rudolfstiftung, Vienna, Austria; ** 1st Medical Department, Krankenanstalt Rudolfstiftung, Vienna, Austria

**Keywords:** tumor, malignancy, mitochondrial disorder, mtDNA, reactive oxidative species

## Abstract

Abstract

Objectives: there are indications that patients with a mitochondrial disorder (MID) develop malignomas or benign tumors more frequently than the general population. The aims of the study were to find out if the prevalence of tumors is actually increased in MID-patients and which of the malignomas or benign tumors are the most frequent. Methods: The charts of MID-patients were retrospectively evaluated for the presence of malign or benign tumors. MID was diagnosed according to the modified Walker-criteria. Results: Among the 475 MID-patients screened for tumors, at least a single malignoma was found in 65 patients (13.7%), and at least a single benign tumor in 35 patients (7.4%). Among those with malignancy, 22 were men and 43 women. Among those with a malignancy, 1 had definite MID, 9 probable MID, and 55 possible MID. The most common of the malignancies was breast cancer, followed by dermatological, gynecological, and gastrointestinal malignancies. The most frequent of the benign tumors was lipoma, followed by pituitary adenoma, meningeomas, carcinoids, and suprarenal adenomas. Compared to the general population, the prevalence of malignancies and of benign tumors was markedly increased. The female preponderance was explained by the frequent maternal inheritance of MIDs. Conclusions: Adult patients with a MID, particularly females, carry an increased risk to develop a malignancy or a benign tumor. Since malignancy is an important determinant for their outcome, these patients should be more accurately screened for neoplasms, not to overlook the point, at which an effective treatment can no longer be provided.

There are indications that patients with a mitochondrial disorder (MID) develop malignomas or benign tumours more frequently than the general population [**1**]. There are also indications that the mutations of the mitochondrial DNA (mtDNA), particularly in oncocytomas, lead to mitochondrial dysfunction, which may directly contribute to cancer progression [**2**]. That mitochondria plays a pathogenetic role in the development of malignancy and is further supported by the fact that cancer cells are generally characterized by the reduction of the oxidative phosphorylation (OXPHOS) together with a marked up-regulation in mitochondrial glycolysis (Warburg effect) [**2**,**3**]. In up to 80% of the various neoplasms mtDNA mutations can be found [**4**]. The aim of this study was to assess the frequency of malignomas and benign tumors in MID patients, to find out which type of tumors may be most frequently found in these patients, and to compare the tumor frequency in MID patients with that of the general population. 

## Patients and methods

Patients with syndromic or non-syndromic MIDs in whom the history was indicative of a tumor were retrospectively evaluated. MIDs were diagnosed according to the modified Walker criteria as definite, probable, and possible [**5**]. A MID was classified as “definite” if the clinical presentation was indicative of a MID and if there was biochemical (deficiency of complex I, II, or IV of the respiratory chain) or genetic evidence of a mitochondrial defect. A MID was classified as “probable” if the clinical presentation was indicative of a MID and if immuno-histological investigations on muscle biopsy showed COX-negative fibers, ragged-red-fibers, SDH-hyper-reactive fibers, or abnormally shaped or structured mitochondria with or without paracrystalline inclusions or glycogen or fat depositions on electron microscopy [**5**]. A MID was classified as “possible” if the clinical presentation suggested a MID (**Table 1**) and if instrumental findings other than a muscle biopsy were indicative of a MID (**Table 2**) [**5**]. The clinical presentation was considered "suggestive" of a MID if at least 3 of the clinical findings listed in Table 1 were present and if additionally at least 3 instrumental findings listed in Table 2 were present or if <3 clinical abnormalities and >10 abnormalities on instrumental investigations were found in a single patient.

**Table 1:**
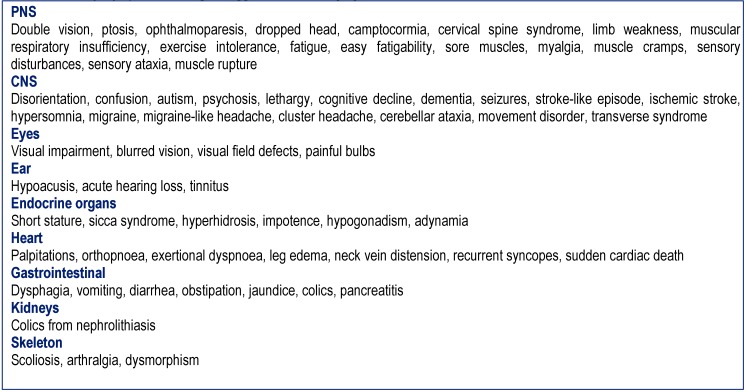
History, symptoms and signs suggestive of a MID [**27**]

**Table 2:**
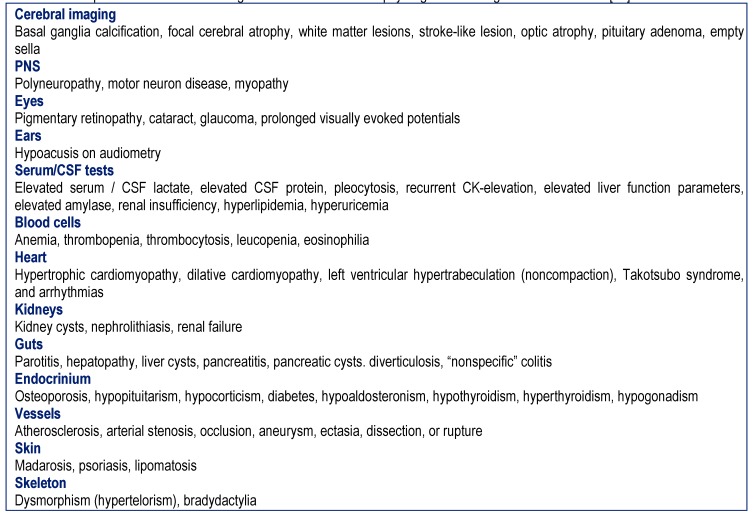
Unexplained instrumental findings other than a muscle biopsy or genetic testing indicative of a MID [**27**]]

Malignancies were counted as such if the report of the histological examination was indicative of a malignancy and if an appropriate treatment was applied. Bowen’s disease of the skin was counted as a malignancy since it is a carcinoma in situ. Benign tumors were defined as those in which histological investigations did not show neoplastic alterations, which presented with typical imaging findings and did not progress at follow-up. A carcinoid was regarded as a benign tumor, if focal or systemic spreading was absent. Unclear cases were classified as such if the patient had a history of surgery indicating a malignoma but no confirmation by histology was available from the charts, if the dignity of the extracted tissue was unclear, or if there was an unclear lesion on imaging of which the dignity could not be assessed and no further information was available. Treatment and outcome were not assessed in this study.

## Results

Altogether, 479 records of patients with a definite, probable or possible MID were screened. Insufficient data were available in 4 patients. Among the remaining 475 patients, at least a single benign or malign tumor was found in 100 patients (21%). At least a single malignoma was found in 65 patients (13.7%), and at least a single benign tumor in 35 patients (7.4%). Twenty cases were classified as unclear. Two consecutively occurring malignomas were found in 6 patients and two benign tumors in 2 patients. Three malignomas were found in a single patient. One malignant and one benign tumor were reported in two patients. One definite malignoma and a questionable second malignoma were found in two patients. Mean age of the 65 patients with at least one malignoma was 75.6y (range: 46 to 93y). Among those with malignancy, 22 were men and 43 female. Mean age of the 35 patients with a benign tumor was 69.2y (range: 34 to 96y). Among those with a benign tumor, 9 were men and 26 were women.

The most frequent among the malignomas was breast cancer (n=17), followed by dermatological malignomas (basalioma, squamous cell carcinoma, melanoma, M. Bowen) (n=12), gynecological tumors (corpus, cervix, ovarial) (n=11), gastrointestinal malignomas (gastric, colon, pancreas) (n=11), and prostate cancer (n=7) (**Table 3**). Other malignomas were more rarely found (**Table 3**). Only one patient with definite MID had a malignoma (thyroid carcinoma), 9 with probable MID and 55 with a possible MID (**Table 3**). The most frequent among the benign tumors were the lipomas (n=12), followed by pituitary adenoma (n=4), meningeomas (n=4), carcinoid tumors (n=4), and suprarenal adenomas (n=4) (**Table 4**). None of the patients with definite MID had a benign tumor but 3 with probable MID and 32 with possible MID. 

**Table 3:**
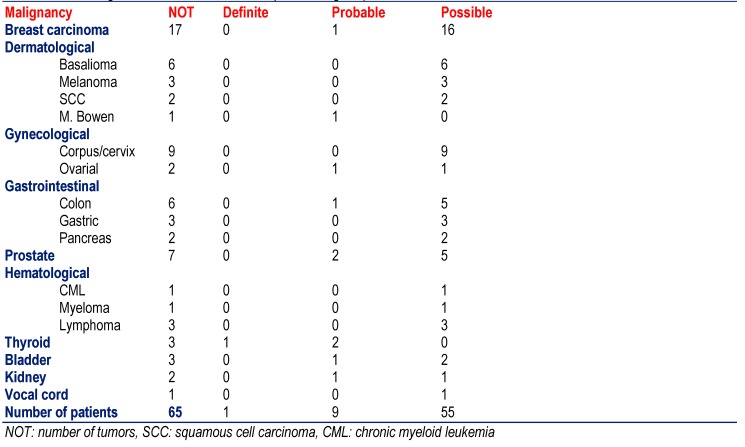
Frequency of malignancies among the 475 included patients with definite, probable, or definite MID (except for the last line the number of malignancies and not those of the patients is given)

**Table 4:**
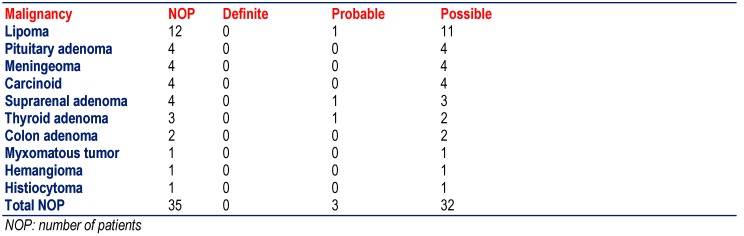
Frequency of benign tumors among the included 475 patients with definite, probable or definite MID (except for the last line the number of malignancies and not those of the patients is given)

**Table 5:**
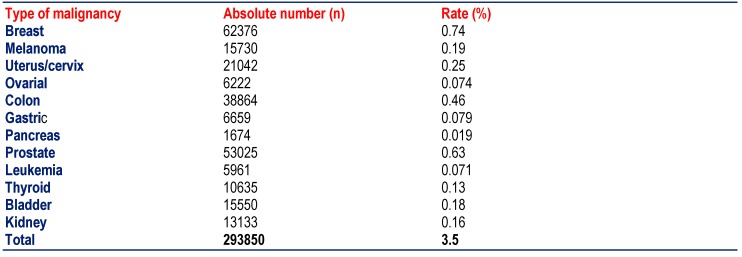
Prevalence of malignancy in Austria at 12/2010. The population in 2010 amounted to 8387742 [**28**]]

37000-38000 patients come down with malignancy every year in Austria. At the end of 2010, the total number of patients alive with malignancy amounted to 293850. Since the population was 8387742 in the year 2010 in Austria, the prevalence of malignancy in the general population was 3.5%. The prevalence of malignancy in MIDs (13.7%) was thus almost four times higher than in the general population. 

## Discussion

This retrospective study showed that one fifth of the adult patients with definite, probable, or possible MID develop a malignant or benign tumor during the disease course. The most frequent of the malignancies in these patients were breast cancer and the most frequent of the benign tumors were lipomas. In malignant as well as benign tumors, there was a striking female preponderance. The frequency of tumors in the investigated cohort was four times higher than in the general population.

There is a number of patients with syndromic or non-syndromic MID in whom malignancy has been described in addition to the mitochondrial defect. These include patients with MELAS-syndrome developing biliary cystadenocarcinoma [**6**] or renal cell carcinoma [**7**], patients with LHON developing acute lymphoblastic leukemia [**8**] or malignant lymphoma [9], patients with maternally inherited diabetes and deafness (MIDD) developing cervical carcinoma [**10**], patients with Leigh syndrome developing liver hepatoblastoma [**11**], patients with depletion syndrome developing hepatocellular carcinoma [**12**], or patients with non-syndromic MID developing thyroid carcinoma [**13**]. MIDs in which benign tumors have been reported include MERRF syndrome, associated with lipoma [**14**-**16**], LHON, associated with pituitary adenoma [**17**], CPEO, associated with lipoma [**18**], or non-syndromic MIDs due to a SDH mutation, associated with paraganglioma or pheochromocytoma [**19**].

The reason why tumors are more prevalent in MID patients compared to the general population is unknown. However, several speculations can be put forward to provide possible explanations for involvement of mitochondrial dysfunction in carcinogenesis [**20**]. Mitochondria are not only responsible for energy production but are also involved in cell proliferation and apoptosis (programmed cell death). They also represent the major site for the generation of oxidative stress in form of reactive oxidative species (ROS) why it has been hypothesized that they play a crucial role in ageing and carcinogenesis [**20**] and directly contribute to cancer progression [2]. Increased ROS production also increases the number of mtDNA mutations [**2**]. Increased number of mtDNA mutations in turn results in increased ROS production, which acts as mutagen or cellular mitogen. In addition to ROS, the mutation rate of the mtDNA may be increased by replication defects, absence of a repair system, and other factors, representing an important further contributor to carcinogenesis [**20**].

More specific explanations for the increased frequency of malignancies in MID patients have been provided by several other studies. There are indications that up-regulation of the OPA1 gene, a mitochondrial fusion-related protein, results in decreased apoptosis [**21**]. Decreased apoptosis could be responsible for the insufficient elimination of depraved cells. On the contrary, there are also indications that mutations associated with Leigh syndrome bolster mitochondria-mediated apoptosis and result in mitochondrial hyperpolarization [**22**]. A further mechanism could be an overexpression of prohibin, an evolutionary conserved protein, which is closely related to malignancy [**23**]. There may also be a reduced expression of the “house-cleaning” enzyme inosine triphosphate pyrophosphohydrolase (ITP), which degrades non-canonical (“regoue”) nucleotides [**24**]. Nucleotide imbalances produced by low ITP-activity may induce mitochondrial dysfunction comprising cell integrity [**24**]. There are also indications that mtDNA mutations produce differences in expression levels of specific nuclear-encoded genes, which are capable to trigger a malignancy phenotype [**25**]. There are also studies that show that mtDNA mutations cause reversible or irreversible changes in genomic DNA methylation of the nuclear DNA [**26**]. The female preponderance in the prevalence of malignomas in MIDs could be explained with the frequent maternal inheritance of the underlying disease or the increased survival of females until advanced age.

It was concluded that adult patients with a MID, particularly females, carry an increased risk of developing malignancy or a benign tumor during the course of the disease. Since malignancy is an important determinant for the outcome, MID patients should be more accurately screened for neoplasms, not to overlook the point, at which an effective treatment can no longer be provided.
